# Enhancing detection of STEC in the meat industry: insights into virulence of priority STEC

**DOI:** 10.3389/fmicb.2025.1543686

**Published:** 2025-02-12

**Authors:** Mai-Lan Tran, Sabine Delannoy, Patrick Fach

**Affiliations:** ‘Pathogenic E. coli’ Unit (COLiPATH) and Genomics platform ‘IdentyPath’ (IDPA), Laboratory for Food Safety, Anses (The French Agency for Food, Environmental and Occupational Health & Safety), Maisons-Alfort, France

**Keywords:** Shiga toxin (Stx) producing *Escherichia coli* (STEC), EHEC (Enterohaemorrhagic *E. coli*), STX, EAE, espK, espV, non-O157, MLG5C.04

## Abstract

Detection of Shiga toxin-producing *Escherichia coli* (STEC) presenting high risk of human infections is challenging. In France, the latest Anses opinion categorized STEC in four groups based on their association with severe forms of clinical infection. STEC strains carrying the *eae* gene, particularly those of serogroups O157, O26, O111, O103, O145, O121, O45 and more recently O80 (top 8 serogroups), are usually monitored worldwide, whereas *eae*-negative STEC strains that are less clinically significant are not surveyed. Screening food enrichment broths with classical genetic markers (*stx*, *eae*) can overestimate the presence of highly virulent STEC, causing needless disruption and costs for food producers. Recently the updated MLG5C reference method introduced additional genetic markers (*espK*, *espV*) in the detection scheme to improve specificity and effectiveness of priority STEC detection in foodstuffs. This study, conducted on beef samples with a new method supporting the regulatory USDA-FSIS MLG5C.04 method, showed that 92% of the *stx*-positive samples carry *stx2* alone or in association with *stx1*. Among the *stx2*-positive samples, *stx2a* and/or *stx2d* subtypes dominate. Introduction of *espK*, *espV* markers on 868 *stx*^+^/*eae*^+^ beef enrichment broths reduced the number of presumptive positive results by 31%, compared to the ISO/TS 13136:2012 reference method. Subsequent analysis of the presumptive positives combining the O-group and the *eae*-subtype provided also a significant reduction of the number of the presumptive positive for the top 8 *eae*-positive STEC serogroups; and showed that O26, O103 and O157 were the most prevalent ones. Regarding the *stx*^+^/*eae^-^* samples, which are proportionally extremely predominant in beef as compared with the *stx*^+^/*eae*^+^ samples, 65% of them were positive for the serogroups monitored in this study (O91, O171, O174, O148, O146, O128 O113 and O104). The high occurrence of serogroup O113 observed in beef samples is not corroborated by the clinical data reported in France. Routine testing of beef samples should be revised to prioritize a hierarchical surveillance system based only on high risk STEC (STEC carrying the *eae* gene) and not on all STEC. This approach would provide Food Business Operators a significant improvement, saving time and costs while maintaining a high level of product safety.

## Introduction

Shiga toxin-producing *Escherichia coli* (STEC) are defined as strains that harbor the *stx* gene, which produces Shiga toxins, but may carry other virulence genes (FAO and WHO, 2018). The Shiga toxins belong to two major types *stx1* further divided into three subtypes (a, c and d) and *stx2* comprising 15 subtypes (a-o) (Lindsey et al., 2023). STEC strains do not carry the “Locus of Enterocyte Effacement” (LEE), a genomic island that includes the *eae* gene encoding the intimin, an adhesion factor to the intestinal mucosa first identified in enteropathogenic *E. coli* (EPEC), and which cause infant diarrhea in developing countries (Olayinka et al., 2024). STEC infections range from mild diarrhea to severe enterohemorrhagic diseases such as hemorrhagic colitis (HC), and hemolytic-uremic syndrome (HUS) (Boerlin et al., 1998; Kobayashi et al., 2013). Although more than 400 STEC serotypes have been isolated from human cases (sporadic and epidemic), only a minority are responsible for most infection cases (Beutin and Fach, 2014). A pathogenic subset called typical enterohemorrhagic *E. coli* (EHEC), defined by the presence of *stx* and *eae* genes, is responsible for enterohemorrhagic disease (Croxen and Finlay, 2010). The most frequent serotypes classified in this group are the top 7 serotypes O157:H7, O26:H11, O111:H8, O103:H2, O145:H28, O121:H19 and O45:H2 (Anses, 2023). Atypical EHEC strains encode *stx* genes but do not carry the LEE. The clinically significant ones belong mainly to O-groups O91, O171, O174, O148, O146, O128, O113, and O104 (Anses, 2023). However, as these are rarely associated with large foodborne outbreaks, they are not currently monitored in foods. While the top 7 serogroups of typical EHEC are closely monitored by U.S. food safety authorities, the EU prioritizes five EHEC serogroups (O157, O26, O111, O103, and O145). In recent years, O80 (*eae*-positive), has emerged as an important serogroup associated with STEC infections in France (Cointe et al., 2018; Cointe et al., 2020) and the French Agency for Food, Environmental and Occupational Health and Safety (Anses) recommends its routine monitoring in foods in the future.

The genetic markers *aaiC* and *aggR* are also used to target some cross-pathotype *E. coli* like the hybrid STEC/EAEC O104:H4 strain that caused a large epidemic in 2011 in both France and Germany, (Bielaszewska et al., 2011; Muniesa et al., 2012). Since then, it has been clearly evidenced that this epidemic strain was not zoonotic (Auvray et al., 2012). More recently, different clones of hybrid STEC of serotype O80:H2 caused HUS cases in Europe (Cointe et al., 2021). These hybrid strains were LEE- and *stx2*-positive (though a particular type of intimin (*eae*-*xi*) and different combinations of *stx2a*, *stx2c* and *stx2d* genes).

Based on French clinical epidemiological and microbiological data and international literature, a categorization of STEC strains has been recently proposed in the latest Anses opinion (Anses, 2023). Clinical isolates were classified in four groups. Group I [(*stx2a* and/or *stx2d*)^+^, (*eae* or *aaiC* and/or *aggR*)^+^] accounts for 84% of the notified HUS and 34% of notified bloody diarrhea. Group II [(*stx2a* and/or *stx2d*)^+^, (*eae* or *aaiC*/*aggR*)^−^] accounts for only 5% of the notified HUS and 6% of notified bloody diarrhea. Group III [(other *stx*-subtypes)^+^, (*eae* or *aaiC*/*aggR*)^+^] is associated with 7% of the notified HUS and 42% of notified bloody diarrhea. Group IV [(other *stx*-subtypes)^+^, (*eae* or *aaiC*/*aggR*)^−^] is reported in only 4% of the notified HUS and 18% of notified bloody diarrhea. French data obtained in Human showed clearly a clinical significance of STEC strains from groups I and III. According to the FAO, STEC strains *stx2a* and *eae*^-^ or *aggR*-positive are at higher risk of causing HUS (FAO and WHO, 2018). In addition, the presence of *stx2d* –alone or in combination with *eae* or *aggR*– is also considered at risk for causing HUS. The National Advisory Committee on Microbiological Criteria for Foods (NACMCF) and the United States Department of Agriculture (USDA) classify STEC carrying *stx2a* with *aggR* or *eae* at high risk of causing severe forms (NACMCF, 2019). These different approaches for assessing STEC pathogenicity nowadays focus on virulence profiles rather than serogroups, moving away from the seropathotype classification (Karmali et al., 2003). Although the serogroup is epidemiologically important for tracking outbreaks, it is insufficient for predicting pathogenicity, especially for strains not fully serotyped.

The ISO/TS 13136 (EU) and MLG5C.03 (US) reference methods (ISO, 2012; USDA-FSIS, 2021) bear the disadvantage to produce many false-positive as the virulence markers (*stx* and *eae*) are found on mobile genetic elements. Thus, other non-pathogenic bacteria and free bacteriophages can carry them as well. These false positives are a global challenge for the regulatory agencies and food producers, causing needless disruption and costs. The USDA Food Safety and Inspection Service (FSIS) has declared six non-O157 STEC serogroups (O26, O45, O103, O111, O121 and O145), known as the “big six,” as adulterants in raw beef products, in addition to *E. coli* O157:H7 (USDA-FSIS, 2012). In 2023, FSIS announced that it began testing all raw beef samples for non-O157 STEC, including raw ground beef, and other beef components (USDA-FSIS, 2023). The MLG5C reference method was updated in 2024 by incorporating recent advancements from the bioMérieux GENE-UP Pathogenic *E. coli* (PEC) kit (USDA-FSIS, 2024). This new approach of the MLG5C.04 includes additional virulence markers like the type III effectors *espK* and *espV* (Delannoy et al., 2013a) enhancing specificity for pathogenic EHEC strains.

This study aims to evaluate the proof of concept of a new method to predict, at the early screening step, beef enrichments broths that are presumptive positives for highly virulent STEC (i.e., strains that carry both the *stx* and *eae* genes, particularly those classified in groups I and III in the latest Anses opinion). This study is not an epidemiological survey and does not reflect the prevalence of STEC or EHEC in the French beef sectors.

## Materials and methods

### Beef samples

Beef samples tested in this study were selected by Interbev (Association Nationale Inter-professionnelle du Bétail et des Viandes) from all production regions of France. They were composed of ground beef and carcasses that were processed by local laboratories with enrichment conditions supporting the growth of non-O157 and O157 STEC (Walker et al., 2024). Then, DNA was extracted from the beef enrichments and tested to determine the *stx/eae* status, as described by Delannoy et al. (2022). Extracted DNA were stored at −20°C until transportation to Anses for PCR analysis.

**Figure 1:**
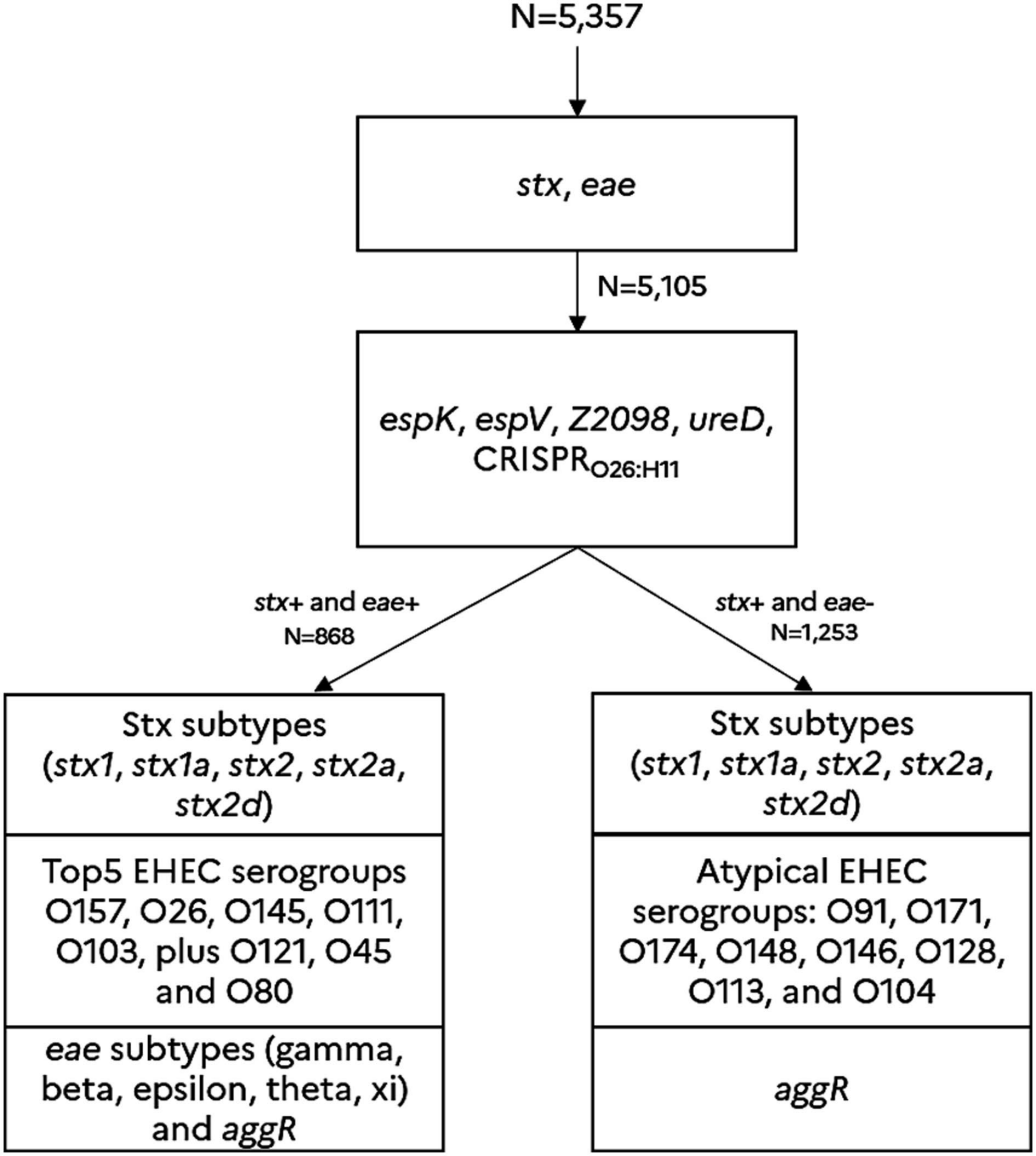
Screening approach for predicting the presence of typical EHEC and atypical EHEC in beef samples. The detection scheme (in line with the new MLG5C.04 reference method) is based on a sequential detection of various genetic markers such as the *stx* genes (including the significant *stx2a* and *stx2d* sub-types), the *eae* gene (including the *eae* sub-types gamma, beta, epsilon, theta and xi), and the molecular markers, *espK*, *espV*, *ureD*, *Z2098*, and CRISPR_O26:H11_. These genetic markers were previously described and validated as hallmarks of virulent STEC (Delannoy et al., 2013a, 2013b, 2015, 2016, 2022). The marker *aggR* is a hallmark of typical enteroaggregative *E. coli*. The genetic markers associated with the main common EHEC O-groups O157 (*eae*-gamma), O145 (*eae*-gamma), O26 (*eae*-beta) O111 (*eae*-thêta), O103 (*eae*-epsilon), O121 (*eae*-epsilon), O45 (*eae*-epsilon) and O80 (*eae*-xi), plus the uncommon EHEC O-groups (O91, O171, O174, O148, O146, O128, O113, and O104) were also tested.

Sample selection was biased to get greater numbers of DNA samples positive for STEC, with a target of 868 samples positive for both *stx* and *eae*. Thus, this sample selection scheme does not reflect the real prevalence of STEC or EHEC in the French beef sectors. However, it is well designed to assess the contribution of the new genetic markers in the reduction in the number of presumptive positive samples after the first phase of screening with *stx* and *eae*. Samples positive for *stx* and *eae* were further PCR tested for the top 5 EHEC serogroups plus O121, O45 and O80, isolation of strains was attempted for confirmation of the top 5 EHEC serotypes regulated in France (O157, O145, O111, O103 and O26). Following the recommendation of the French ministry of agriculture, the appropriate sanitary measures were taken in positive cases of EHEC top 5. Moreover, regarding samples that were positive for *stx* only, they were further PCR tested for the following atypical EHEC O-groups: O91, O171, O174, O148, O146, O128, O113, and O104 (Figure 1).

### High-throughput real-time PCR

As the high-throughput real-time PCR system relies on microfluidic PCR (PCR reaction taking on place on few nanoliters), a pre-amplification is required to guaranty the sensitivity of the method (Michelet et al., 2014). DNA extracts were processed by high-throughput real-time PCR using the Biomark™ real-time PCR system (Standard BioTools, USA) as described previously in an equivalent study performed on dairy products (Delannoy et al., 2022). Primers and probes used in high-throughput real-time PCR were those previously described by Delannoy et al. (2022) or were designed for this study. Table 1 reports all oligonucleotides used for this study.

**Table 1:** Primers and probe sequences used in this study.

Primer or probe^a^	Sequence 5′ → 3’^b^
*stx1*_F^c^	TTTGTYACTGTSACAGCWGAAGCYTTACG
*stx1*_R^c^	CCCCAGTTCARWGTRAGRTCMACRTC
*stx1*_P^c^	CTGGATGATCTCAGTGGGCGTTCTTATGTAA
*stx1a*_F^l^	ATGGACAAGACTCTGTTCGTGTA
*stx1a*_R^l^	AATTCAGTATTAATGCCACGCTT
*stx1a*_P^l^	CCAGAATTGCATTAATGCTTCCAAAAGAA
*stx2*_F^c^	TTTGTYACTGTSACAGCWGAAGCYTTACG
*stx2*_R^c^	CCCCAGTTCARWGTRAGRTCMACRTC
*stx2*_P^c^	TCGTCAGGCACTGTCTGAAACTGCTCC
*stx2a*_F^l^	TTCTGTTAATGCAATGGCGGCG
*stx2a*_R^l^	CCAGTATTCTTTCCCGTCAACCTTC
*stx2a*_P^l^	AATGTGTCATCCTCATTATACTTGG
*stx2d*_F	TGGGAAAGTAATACAGCAGCAG
*stx2d*_R	TCTTCTTGATACTTAACTGCTTTATTC
*stx2d*_P	CCCGTTGTATATAAAGACTGTGACTTTCTGTTCA
*stx2dg*_F	AGCAGCAGCCTTTCTGAACAG
*stx2dg*_R	CCGCCATAAACATCTTCTTCATACTTAA
*stx2dg*_P^n^	CAACGGGTGAATAA
*eae*_F^d^	CATTGATCAGGATTTTTCTGGTGATA
*eae*_R^d^	CTCATGCGGAAATAGCCGTTA
*eae*_P^d^	ATAGTCTCGCCAGTATTCGCCACCAATACC
*eae*-beta_F^d^	GGTGATAATCAGAGTGCGACATACA
*eae*-beta_R^d^	GGCATCAAAATACGTAACTCGAGTAT
*eae*-beta_P^d^	CCACAGCAATTACAATACTACCCGGTGCA
*eae*-gamma_F^d^	GACTGTTAGTGCGACAGTCAGTGA
*eae*-gamma_R^d^	TTGTTGTCAATTTTCAGTTCATCAAA
*eae*-gamma_P^d^	TGACCTCAGTCGCTTTAACCTCAGCC
*eae*-epsilon_F^d^	ATACCCAAATTGTGAAAACGGATA
*eae*-epsilon_R^d^	CACTAACAACAGCATTACCTGCAA
*eae*-epsilon_P^d^	CCAGATGTCAGTTTTACCGTAGCCCTACCA
*eae*-theta_F^d^	TGTTAAAGCACCTGAGGTTACATTTT
*eae*-theta_R^d^	TCACCAGTAACGTTCTTACCAAGAA
*eae*-theta_P^d^	TCAACCTTGTTGTCAATTTTCAGTCCATCA
*eae*-xi_F	ACGATGCTGAAGCAATATGTAGAAC
*eae*-xi_R	CGCTCCCCATTTATTATACACG
*eae*-xi_P	TTTAACTCATTCGTAGATAGCGGTAAACGGC
*espK*_F^e^	GCAGRCATCAAAAGCGAAATCACACC
*espK*_R^e^	TCGTTTGGTAACTGTGGCAGATACTC
*espK*_P^e^	ATTCAGATAGAAGAAGCGCGGGCCAG
*espV*_F^e^	TCAGGTTCCTCGTCTGATGCCGC
*espV*_R^e^	CTGGTTCAGGCCTGGAGCAGTCC
*espV*_P^e^	CTTGCAACACGTTACGCTGCCGAGTATT
*Z2098*_F^f^	CTGAAAAGAGCCAGAACGTGC
*Z2098*_R^f^	TGCCTAAGATCATTACCCGGAC
*Z2098*_P^f^	TAACTGCTATACCTCCGCGCCG
*ureD*_F^e^	GCAATAATTGACTCTGATTGCC
*ureD*_R^e^	GCTGCTGCGGTAAAATTTACT
*ureD*_P^e^	TACGCTGATCACCATGCCTGGTGC
*aggR*_F ^m^	GCCTAAAGGATGCCCTGATG
*aggR*_R ^m^	GACCAATTCGGACAACTGCAA
*aggR*_P ^m,n^	CATCTACTTTTGATATTCCGTAT
CRISPR_O26_F^g^	AAACCGATCTCCTCATCCTC
CRISPR_O26_R^h^	ATCAACATGCAGCGCGAACG
CRISPR_O26_P^g^	CCAGCTACCGACAGTAGTGTGTTCC
rfbE_O157_-F^c^	TTTCACACTTATTGGATGGTCTCAA
rfbE_O157_-R^c^	CGATGAGTTTATCTGCAAGGTGAT
rfbE_O157_-P^c^	AGGACCGCAGAGGAAAGAGAGGAATTAAGG
wzx_O26_-F^c^	CGCGACGGCAGAGAAAATT
wzx_O26_-R^c^	AGCAGGCTTTTATATTCTCCAACTTT
wzx_O26_-P^c^	CCCCGTTAAATCAATACTATTTCACGAGGTTGA
wzx_O103_-F^i^	CAAGGTGATTACGAAAATGCATGT
wzx_O103_-R^i^	GAAAAAAGCACCCCCGTACTTAT
wzx_O103_-P_i_	CATAGCCTGTTGTTTTAT
wbdl_O111_-F^c^	CGAGGCAACACATTATATAGTGCTTT
wbdl_O111_-R^c^	TTTTTGAATAGTTATGAACATCTTGTTTAGC
wbdl_O111_-P^c^	TTGAATCTCCCAGATGATCAACATCGTGAA
wzx_O121_-F^j^	TGGTCTCTTAGACTTAGGGC
wzx_O121_-R^j^	TTAGCAATTTTCTGTAGTCCAGC
wzx_O121_-P^j^	TCCAACAATTGGTCGTGAAACAGCTCG
wzx_O45_-F^j^	TACGTCTGGCTGCAGGG
wzx_O45_-R^j^	ACTTGCAGCAAAAAATCCCC
wzx_O45_-P^j^	TTCGTTGCGTTGTGCATGGTGGC
wzy_O145_-F^k^	ATATTGGGCTGCCACTGATGGGAT
wzy_O145_-R^k^	TATGGCGTACAATGCACCGCAAAC
wzy_O145_-P^k^	AGCAGTGGTTCGCGCACAGCATGGT
wzx_O80_-F^l^	CAGTTATACCGATCCTTAATTTACAAGGA
wzx_O80_-R^l^	GCTTACAAAAGACACTGGAATTATAATTCC
wzx_O80_-P^l^	CGCAGGGTTATCGATTTTGGGTGCTACT
wzy_O91_-F^c^	CGATTTTCTGGAATGCTTGATG
wzy_O91_-R^c^	CAATACATAGTTTGATTTGTGTTTAAAGTTTAAT
wzy_O91_-P^c^	CCTGGGTTGTTAGGAACAATTTCAGCACTTC
wzx_O171_-F	ACCAATTTGTCTTCTCCCTAGCAT
wzx_O171_-R	ATGTGCCAATGAACTCATATTTCTCT
wzx_O171_-P	TATTTCATTCCTCCTGTTTTTTTCGTGGCA
wzx_O174_-F	CCGCTTACTGGGAAGCATTTA
wzx_O174_-R	ACACTGCAATGGAAGTTAAGGCTATA
wzx_O174_-P	ATGGGGCAGAACTTAGCAAAACCTTATTGGC
wzy_O148_-F	GGGATTGGCAGCCTTTAATCT
wzy_O148_-R	CAAACCTCCTTAAATGGATACAAAAGT
wzy_O148_-P	TATGTTGAAAAGAATGTCACGCTCCCCG
wzx_O146_-F	GGCCACAAAGATAGCAACAAGATA
wzx_O146_-R	CAGGCAACAGGTAAAATCAAAATCTA
wzx_O146_-P	TTCAGCAACATTACTCCACCAACCACCA
wzx_O128_-F	GCAACCCCAATAGCAAAAGCT
wzx_O128_-R	TTTTGCGAAAAGAAAATGAAGGT
wzx_O128_-P	AACAACCTGAACAAGACGATCGAAAAAGCC
wzy_O113_-F^c^	GAGCGTTTCTGACATATGGAGTGA
wzy_O113_-R^c^	TTGCTATAAATGGAAGCCATTCTTT
wzy_O113_-P^c^	TGCATGAAATGTTTAAATGCAGCGGGT
wzx_O104_-F^j^	TGTCGCGCAAAGAATTTCAAC
wzx_O104_-R^j^	AAAATCCTTTAAACTATACGCCC
wzx_O104_-P^j^	TTGGTTTTTTTGTATTAGCAATAAGTGGTGTC

^a^F, forward primer; R, reverse primer; P, probe. ^b^Probes were labeled with either 6-HEX or 6-FAM and BHQ1 (Black Hole Quencher). ^c^Oligonucleotide described by Perelle et al. (2004), ^d^Oligonucleotide described by Nielsen and Andersen (2003), ^e^oligonucleotide described by Delannoy et al. (2013a), ^f^oligonucleotide described by Delannoy et al. (2013b), ^g^oligonucleotide described by Delannoy et al. (2012a), ^h^oligonucleotide described by Delannoy et al. (2015), ^i^oligonucleotide described by Perelle et al. (2005), ^j^oligonucleotide described by Bugarel et al. (2010), ^k^oligonucleotide described by Fratamico et al. (2009), ^l^oligonucleotide described by Delannoy et al. (2022), ^m^oligonucleotide described by Delannoy et al. (2012b), ^n^Probe was labeled with minor groove binder (MGB). To be recorded as positive for *stx2d*, a beef enrichment should be simultaneously PCR positive for both *stx2d* and *stx2dg*.

### Presumptive positives for STEC belonging to groups I and III

Regarding the determination of presumptive positive samples for group I and III, three methods were explored and are defined as followed: method A (*stx*, *eae*, *espK*/*Z2098*/CRISPR_O26:H11_), method B (*stx*, *eae*, *espK*/*espV*/CRISPR_O26:H11_) and method C (*stx*, *eae*, *espK*/*ureD*/CRISPR_O26:H11_). A sample was considered presumptive positive if it tested positive for *stx*, *eae*, and at least one of the additional targets specified in each method. For example, a presumptive positive for method A was defined as a sample that tested *stx*+, *eae*+, and *espK*/*Z2098*/CRISPR_O26:H11_+ (meaning positive for at least one of the three targets *espK*, *Z2098*, CRISPR_O26:H11_). The same principle applies to methods B and C, where *espV* or *ureD* serve as the respective additional targets.

In a second step, all DNA samples *stx^+^*/*eae^+^* (i.e., presumptive positives for group I and III) were further tested by real-time PCR for the main typical EHEC O-groups (O157, O26, O145, O111, O103, O145, O121, O45 and O80) and their corresponding *eae*-variants (*eae*-gamma, −beta, −epsilon, −theta, −xi subtypes). A presumptive positive recorded in this second screening was a sample *stx*^+^/*eae*^+^ that tested positive for the appropriate association O-group/*eae*-subtype.

The first screening with the new EHEC markers (methods A, B and C) reports the number of presumptive positives for all typical EHEC serotypes and not only for the top 7 EHEC serotypes. The second screening, as based on the combination of the O-group/*eae* type reports the number of presumptive positives for the top 7 EHEC serogroups plus O80, which are usually monitored worldwide. Data presented in this study showed the number of presumptive positives after the first screening (New EHEC markers) and the second screening step (O-groups/*eae*-types). Data were compared with the ISO/TS 13136 reference method (ISO, 2012).

### Presumptive positives for STEC belonging to groups II and IV

Regarding the determination of presumptive positive samples for EHEC of groups II and IV, all DNA samples *stx^+^*/*eae^-^* were further PCR tested for the main atypical EHEC O-groups (O91, O171, O174, O148, O146, O128, O113, and O104).

### Statistical analysis

Statistical analyses were performed on R Studio version 2021.09.0. Two-proportions *z*-tests (two-tailed or one tailed) were used to compare proportions of presumptive positives between the ISO reference method and the new approach (methods A, B, and C). The statistical tests were performed with *α* of 5%.

## Results

### Quality control, number of samples and PCR data points included in the dataset

DNA extracts (*n* = 5,357) from beef were sent to Anses. The *stx*/*eae* status as determined by the local laboratories and Anses was identical for 5,105 DNA extracts. These samples having the same *stx*/*eae* status by conventional real-time PCR and by microfluidics real-time PCR were further used in this study. DNA extracts analysed in this study were either positive for *stx* alone (*n* = 1,253), positive for *stx* and *eae* (*n* = 868), positive for *eae* alone (*n* = 1,232), or negative for both *stx* and *eae* (*n* = 1,752). The study as a whole represents 225,504 real-time PCR determinations, which served to consolidate the results presented in this manuscript.

### Screening samples for stx1, stx2, stx2a, and stx2d subtypes

Among the DNA samples tested, a total of 2,121 DNA extracts were *stx*-positive (1,253 positive for *stx* only and 868 positive for both *stx* and *eae*). The data showed that the distribution of the *stx1* and *stx2* genes is quite different (Table 2). It is remarkable that only 8% of the *stx*-positive samples are positive for *stx1* only. The *stx2* gene was significantly more prevalent with 67% of the samples positive for *stx2* only and 25% positive for *stx1* and *stx2* (*p* < 0.05). As a whole, 92% of the *stx*-positive samples are thus positive for *stx2* alone or in association with *stx1*. Among the 1,955 *stx2*-positive samples, 93% are positive for at least *stx2a* and/or *stx2d*, showing the high prevalence of these two subtypes in beef samples. The other *stx2* positive samples (approximately 6%) were positive for *stx2*-subtypes other than *stx2a* and *stx2d*.

**Table 2:** Distribution of *stx1* and *stx2* genes in 2,121 *stx*-positive beef samples.

*stx* category	N	*stx*-subtypes	*N*
*stx1* only	166	*stx1a* ^+^	160
		*stx1a^−^*	6
*stx2* only	1,416	(*stx2a* and/or *stx2d*)^+^	1,310
		(*stx2a* and/or *stx2d*)^−^	106
*stx1* + *stx2*	539	*stx1a*^+^, (*stx2a* and/or *stx2d*)^+^	420
		*stx1a*^+^, (*stx2a* and/or *stx2d*)^−^	106
		stx*1a*^−^, (*stx2a* and/or *stx2d*)^+^	8
		stx*1a*^−^, (*stx2a* and/or *stx2d*)^−^	5

Beef samples positive for *stx1* only (*N* = 166) include 160 samples positive for *stx1a* only and 6 for *stx1* subtypes other than *stx1a*. Beef samples positive for *stx2* only (*N* = 1,416) include 1,310 samples positive for (*stx2a* and/or *stx2d*) only and 106 for *stx2* subtypes other than *stx2a* and *stx2d*. Among the 539 beef samples positive for both *stx1* and *stx2*, 420 samples were *stx1a^+^* and (*stx2a* and/or *stx2d*)^+^, 106 *stx1a^+^* and (*stx2a* and/or *stx2d*)^−^, 8 *stx1a^-^* and (*stx2a* and/or *stx2d*)^+^ and 5 *stx1a^-^* and (*stx2a* and/or *stx2d*)^−^.

All the 2,121 *stx*-positive samples were also categorized in groups I to IV based on the *stx2a* and *stx2d* real-time PCR results and the detection of *eae* and *aggR*. Overall, group I [(*stx2a* and/or *stx2d*)^+^, *eae*^+^] comprised 724 samples, group II [(*stx2a* and/or *stx2d*)^+^, *eae*^-^] comprised 1,102 samples, group III (other *stx*^+^, *eae*^+^) comprised 144 samples and group IV (other *stx*^+^, *eae*^-^) comprised 151 samples. It is remarkable to note that all *stx*^+^ samples tested negative for *aggR* (data not shown).

### Screening stx-and eae-positives (i.e., samples classified in groups I and III) for espK, espV, Z2098, ureD and CRISPR_O26:H11_

**Figure 2:**
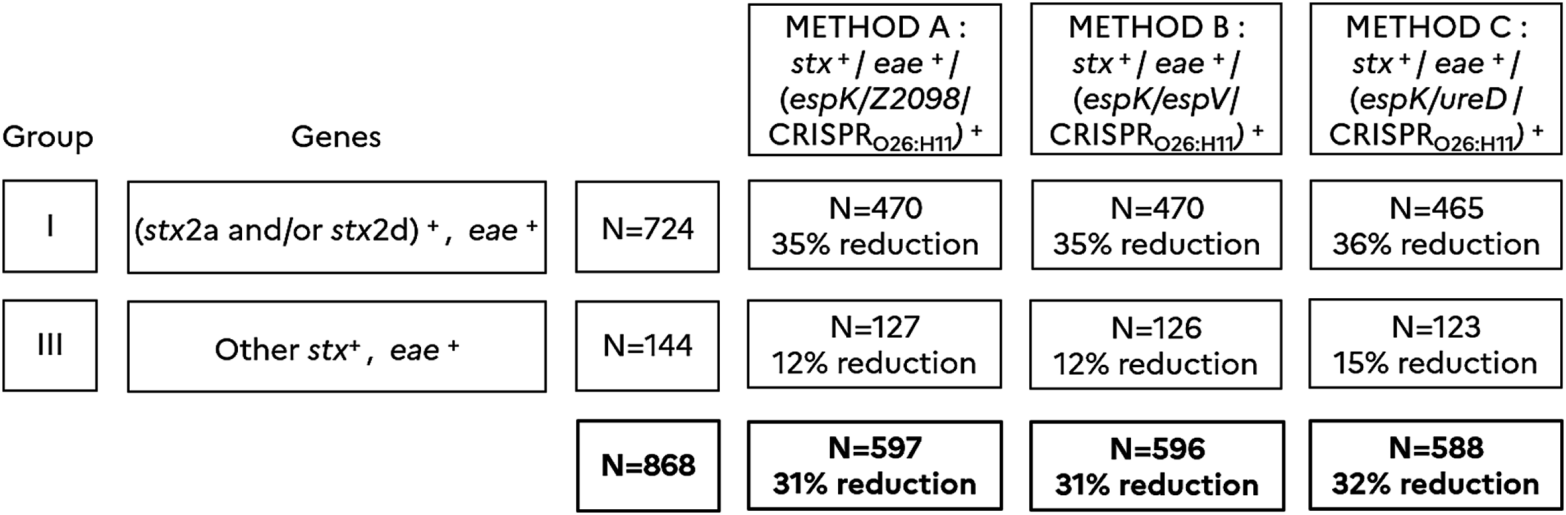
Classification of 2,121 *stx*-positive samples according to Anses opinion (2023) and comparison of methods A, B and C. (*stx2a* and/or *stx2d*)^+^ includes samples giving a positive result for *stx2a* and/or *stx2d*. Other *stx*^+^ includes samples giving a positive result for *stx* subtypes other than *stx2a* and *stx2d*. *eae*^+^ includes samples giving a positive result for *eae*. (*espK*/*Z2098*/CRISPR_O26:H11_)^+^ includes samples giving a positive result for at least one of the markers *espK*, *Z2098*, or CRISPR_O26:H11._ (espK/espV/CRISPR_O26:H11_)^+^ includes samples giving a positive result for at least one of the markers *espK*, *espV*, or CRISPR_O26:H11._ (*espK*/*ureD*/CRISPR_O26:H11_)^+^ includes samples giving a positive result for at least one of the markers *espK*, *ureD*, or CRISPR_O26:H11._ Samples are categorized in group I (*N* = 724) and III (*N* = 144) according to the Anses opinion. The percentage of reduction in the number of presumptive positives is calculated for each method A, B and C.

A total of 868 samples *stx*^+^/*eae*^+^ were investigated with methods A, B and C and the number of presumptive positives was reported for each method. In total, 597 presumptive positives were recorded according to method A, 596 with method B and 588 with method C. Alternate methods of pre-screening (methods A, B, and C) that include in addition to *stx*/*eae*, the detection of five novel markers (*espK*, *espV*, *Z2098*, *ureD*, and CRISPR_O26:H11_) allowed a significant reduction in the number of presumptive positives, with a reduction rate of approximately 31–32% with regard to the ISO/TS 13136:2012 reference method (Table 3). As shown in Figure 2, the reduction of the number of presumptive positives obtained with methods A, B and C is significantly higher for samples classified in group I (35–36% reduction) than in group III (12–15% reduction).

**Table 3:** Reduction of the number of presumptive positives by introducing new EHEC markers in the first screening phase of the ISO/TS 13136 reference method.

	METHOD A: *stx* ^+^/*eae* ^+^/(*espK/Z2098*/ CRISPR_O26:H11_) ^+^	METHOD B: *stx* ^+^/*eae* ^+^/(*espK/espV*/ CRISPR_O26:H11_) ^+^	METHOD C: *stx* ^+^/*eae* ^+^/(*espK/ureD*/CRISPR_O26:H11_) ^+^
*N*	597	596	588
*p*-value^a^	2.004e-14	1.581e-14	2.286e-15
Reduction of the number of presumptive positives	31%	31%	32%
*p*-value^b^	1.002e-14	7.905e-15	1.143e-15

^aTwo-tailed two-proportions z-test, α = 0.05. The proportion of presumptive positives with methods A, B and C was significantly different from the ISO/TS 13136 reference method when p < 0.05. bOne-tailed two-proportions z-test, α = 0.05. The proportion of presumptive positives with the ISO/TS 13136 reference method was significantly greater than methods A, B and C when p < 0.05. A total of 5,105 beef samples were tested and 868 were positive for stx and eae.^

### Screening stx-and eae-positives (i.e., samples classified in groups I and III) for eae subtypes gamma, beta, epsilon, theta and xi and correlation with the serogroups O157, O145, O121, O103, O111, O26, O45, and O80

As methods A, B and C gave equivalent results, we chose method B, that is in line with the MLG5C.04, as an alternate method performing well to further analyze samples of groups I and III for the O-groups and the *eae* subtypes. Based on (1) the data obtained with the pre-screening method B, and (2) the data on the correlation between the presence of the O-antigen markers and the *eae*-subtypes, we could compare the results of this alternate method with the conventional ISO/TS 13136:2012 reference method which takes into account only the *stx*, *eae* and the O-antigen markers. Results were expressed for the top 7 EHEC serogroups plus serogroup O80 (top 8) that has recently emerged in Europe.

**Figure 3:**
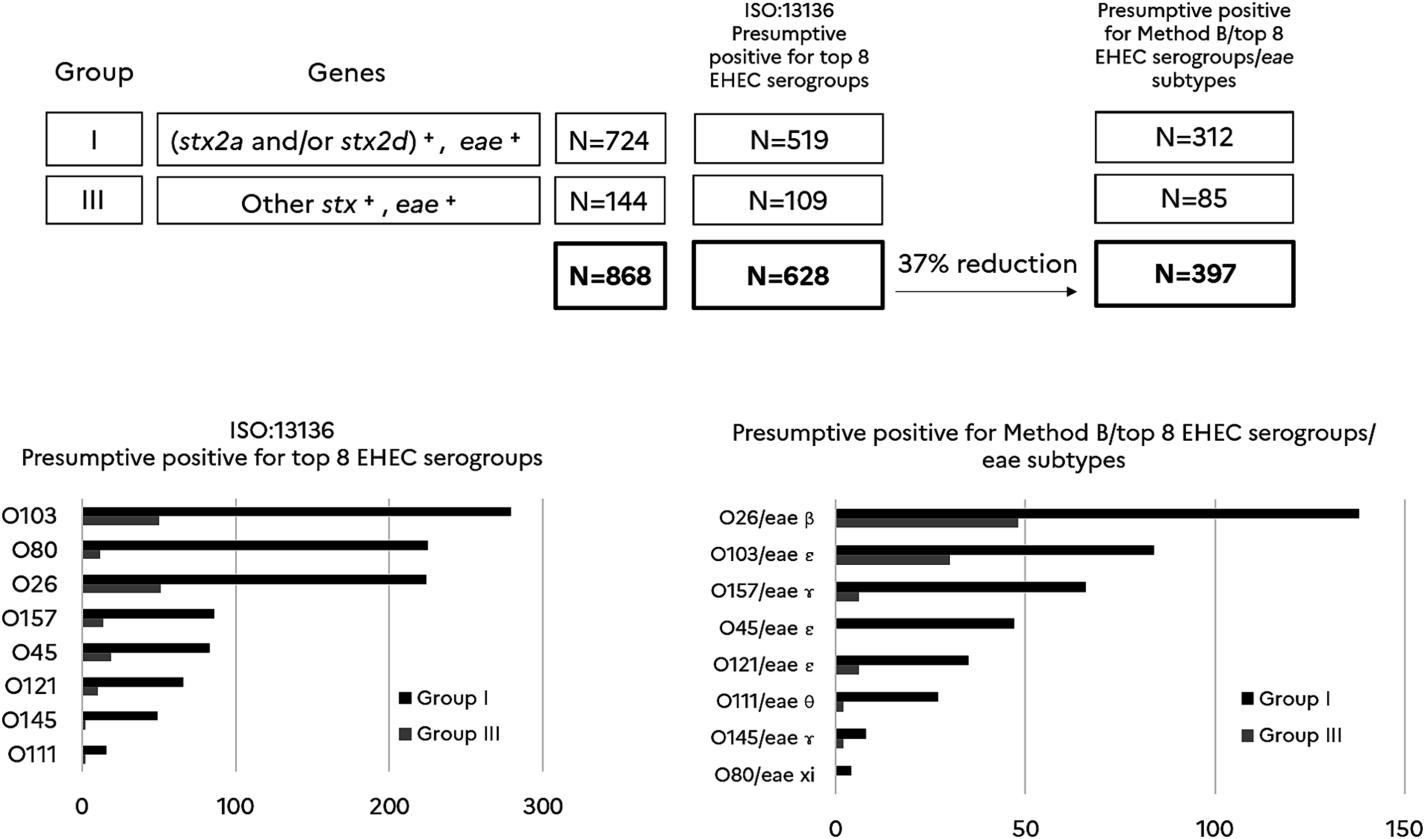
Comparison of presumptive positives for typical EHEC serogroups (top 8) using the ISO/TS13136:2012 reference method vs the new approach. (*stx2a* and/or *stx2d*)^+^ includes samples giving a positive result for *stx2a* and/or *stx2d*. Other *stx*^+^ includes samples giving a positive result for *stx* subtypes other than *stx2a* and *stx2d*. *eae*^+^ includes samples giving a positive result for *eae*. Samples are categorized in group I (*N* = 724) and III (*N* = 144) according to the Anses opinion. *N* = 628 is the number of presumptive positives for the top 8 EHEC serogroups according to the ISO:13136 reference method. *N* = 397 is the number of presumptive positives recorded by combining Method B, the top 8 EHEC serogroups and their corresponding *eae* subtype. The percentage of reduction in the number of presumptive positives is calculated for the new method when compared with the ISO reference method (*p* < 0.05). For each method, the histograms report the number of presumptive positives for each O-group and for samples categorized in groups I and III.

As shown in Figure 3 the number of presumptive positives for the top 8 typical EHEC serogroups identified with the ISO/TS 13136:2012 reference method is higher (628 samples) than those identified with the new approach including a pre-screening with method B and subsequent analysis with the combination of the O-group and the *eae*-subtype (397 samples). Thus, the new approach significantly reduced the number of the presumptive positive top 8 EHEC serogroups by 37% compared to the conventional ISO method (*p* < 0.05).

It is also noteworthy that the association of the O-groups with *eae*-subtypes affects the ranking of the top 8 typical EHEC serogroups with regard to the categorization obtained with the ISO reference method. The greatest selective effect was observed for O80. The association with *eae*-xi and O80 allowed discarding most of the presumptive samples for EHEC O80, showing that non-EHEC O80 are probably more prevalent in beef than EHEC O80.

### Screening stx-positive and eae-negative samples (i.e., samples classified in groups II and IV) for the serogroups O91, O171, O174, O148, O146, O128, O113 and O104

**Figure 4:**
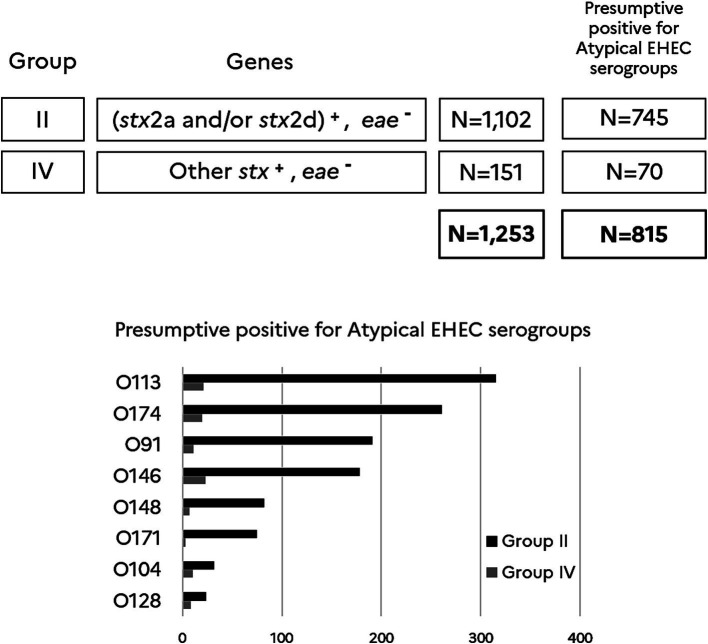
Atypical EHEC top 8 serogroups in *stx*^+^/*eae^-^* beef samples. (*stx2a* and/or *stx2d*)^+^ includes samples giving a positive result for *stx2a* and/or *stx2d*. Other *stx*^+^ includes samples giving a positive result for *stx* subtypes other than *stx2a* and *stx2d*. *eae-includes* samples giving a negative result for *eae*. Samples are categorized in group II (*N* = 1,102) and IV (*N* = 151) according to the Anses opinion. *N* = 815 is the number of presumptive positives for the top 8 atypical EHEC serogroups recorded in this study. The number of presumptive positives for each O-group and for samples categorized in groups II and IV is reported in the histogram.

With the objective to get an estimation of uncommon and atypical EHEC and their most prevalent serogroups, the *stx*^+^/*eae^-^* samples, which are categorized in groups II and IV, were tested for the French clinically significant top 6 atypical EHEC serogroups (O91, O171, O174, O148, O146, O128) plus serogroups O113 and O104. As shown in Figure 4, the data indicated that most of the atypical EHEC presumptive samples are categorized in group II (1,102 samples) and little in group IV (151 samples). Serogroup O113 highly dominates and is followed with a decreasing occurrence by serogroups O174, O91/O146, O148, O171, O104 and O128. One could notice in addition that 35% of the samples classified in groups II and IV tested negative for all the atypical EHEC serogroups monitored in this study (O91, O171, O174, O148, O146, O128, O113, and O104).

## Discussion

It remains difficult to fully define human pathogenic STEC or identify virulence factors for STEC that absolutely predict the potential to cause human disease (EFSA and ECDC, 2021). However, data analysis of the French and European epidemiological studies underlines the relative importance of some virulence factors/markers. In the latest Anses opinion (Anses, 2023), the characteristics of strains isolated from cases of STEC infections are as follows: (i) the *stx2* gene is found in 82% of cases of infections and 95% of cases of HUS; (ii) the *eae* gene is found in 80% of cases of infections and 90% of cases of HUS; (iii) the *stx* sub-types mostly associated with HUS are *stx2a* (58%) and *stx2d* (31%); (iv) three virulence profiles are responsible for more than 80% of HUS: [*stx2a*^+^/*eae*^+^], [*stx2d*^+^/*eae*^+^] and [*stx1a*^+^/*stx2a*^+^/*eae*^+^]; (v) the top 5 serogroups (O157, O26, O103, O145 and O111) and O80 represent 80% of cases of HUS in France. Regarding the clinical isolates that were *eae*-negative, the most frequently found serogroups are O146, O91 and O128.

Detection of EHEC in foods remains particularly challenging. In the present study, we designed and optimized a molecular risk assessment approach in beef samples that is in line with the MLG5C.04 reference method. This approach aims to provide a significant reduction in false positives while ensuring alignment with the latest Anses recommendations (Anses, 2023) that pointed out STEC of groups I and III as higher risk. For food business operators (FBO) it is important to improve the current detection methods with the aim of increasing the percentage of batches released at the end of the first screening step and of reducing the number of confirmations on isolated colonies while maintaining a high (or even higher) level of product safety.

In this study on beef samples collected in France, we showed that among the *stx* positive enriched beef samples the occurrence of *stx2* is very high (*p* < 0.05). Indeed, it is remarkable that 92% of the *stx* positive enriched beef samples carry *stx2* alone (67%) or in association with *stx1* (25%). Moreover, the *stx2* sub-typing scheme allowed to estimate that 93% of the *stx2* positive samples are also positive for at least *stx2a* and/or *stx2d*. After testing the *stx* ^+^ samples for the *eae* gene, one could categorize them into groups I to IV according to the latest Anses opinion (Anses, 2023). Among the *stx* ^+^ beef samples, those categorized in group II dominate over the other groups. To refine the analysis of beef enrichments classified in groups I and III, additional biomarkers were tested in complement to the *stx* and *eae* genes. This new panel of genes included genetic markers identified previously (Delannoy et al., 2013a, 2013b) as highly associated with pathogenic STEC strains (*stx*+, *eae*+), detecting the top 7 EHEC together with many other serotypes than the top 7 of typical EHEC, offering then the possibility for identifying new emerging typical EHEC strains (Delannoy et al., 2013a, 2013b). Conversely, these genetic markers were very rarely (1.1–3.4%) associated with STEC and with non-pathogenic *E. coli*. Introduction of these markers in the detection scheme provided a significant reduction in the number of *stx*+/*eae* + beef samples that require a second screening step for serogroup determination. Moreover, as it relies on the detection of *espK*/*espV* it is also consistent with the updated MLG5C.04 reference method (USDA-FSIS, 2024).

Hence, a 31% reduction in the number of presumptive positive was achieved by using the alternate method A (*stx*/*eae*/*espK*/*Z2098*) or method B (*stx*/*eae*/*espK*/*espV*), and 32% with method C (*stx*/*eae*/*espK*/*ureD*) when they were combined with the CRISPR_O26:H11_ PCR assay. This CRISPR based PCR assay was introduced to detect a minor O26 lineage of EHEC that emerged 10 years ago in France but which declined and is no longer detected in humans (Delannoy et al., 2015). The three methods performed well and gave equivalent levels of selectivity to get a more precise risk assessment. As it is in line with MLG5C.04 reference method, we chose method B (*stx*/*eae*/*espK*/*espV*) in association with the CRISPR_O26:H11_ PCR assay, to narrow down the EHEC screening step in beef samples and to go further for EHEC serogroups determination. The new approach including a pre-screening with method B and subsequent analysis combining the O-group and the *eae*-subtype provided a 54% reduction in the number of the presumptive positive top 7 EHEC plus EHEC O80 (top 8 EHEC serogroups) as compared with a single screening for *stx*^+^/*eae*^+^. Presumptive positive samples for the top 8 EHEC serogroups accounted for 43% of samples of group I and 59% of group III. The first three serogroups of typical EHEC in presumptive positive samples are O26, O103 and O157.

Regarding beef samples (*stx*^+^, *eae*^-^) classified in groups II and IV, 88% were in group II and 12% in group IV. Group II, which appears extremely predominant in the beef industry, remains very rarely associated with STEC infections in France (Anses, 2023). Presumptive positive samples for the atypical top 8 EHEC serogroups accounted for 68% of samples of group II and 46% of group IV. The decreasing occurrence of atypical EHEC serogroups is the following: O113, O174, O91/O146, O148, O171, O104 and O128. Serogroup O113 is the most common, whereas serogroup O104 circulates poorly. The high occurrence of serogroup O113 in beef samples is not corroborated by the clinical data reported in the latest Anses opinion (Anses, 2023). As there is no correlation between the epidemiological data observed from the clinical side and the beef industry, we should rethink about the routine testing of raw meat to prioritize a hierarchical surveillance system, which would be based only on high risk STEC and not on all STEC. Given the data recorded in clinical samples in France and those obtained in this study, it makes sense for the routine surveillance of raw meat to focus only on highly virulent STEC of groups I and III (*stx*^+^, *eae*^+^). Here, we showed that the introduction of the new EHEC markers *espK*/*espV* in the detection scheme (at the first screening step on enriched samples) would certainly provide the beef industry a selective and reliable method for tracking the regulated top 7 EHEC serogroups together with other *eae*-positive STEC serotypes that may emerge in the future. The multiplication of genetic markers that must be included in the detection scheme may be one limitation of this approach as it requires optimization of multiplex PCRs for high throughput routine testing. Multiplication of genetic markers will also increase the cost of the routine analysis for screening highly virulent STEC in beef. Another limitation is that one could never exclude the emergence of new virulent STEC strains that would fail to be detected with these markers. The method developed as part of this project has nonetheless the potential to be used to prioritize STEC risk in raw meat, as although Shiga toxin is essential to STEC pathogenesis, not all STEC strains are systematically associated with disease in humans. The FBO need for their own controls an approach that focus on the higher risk STEC. The approach described here aligns with the updated USDA-FSIS MLG5C.04 method while adapting to the complexities of STEC virulence and epidemiology in the food industry.

## Conclusion

Here we propose an optimized testing scheme that includes the detection of additional virulence markers characterizing virulent STEC. By providing a reduction of more than 30% in the number of presumptive positives, this method improves the specificity and effectiveness of STEC detection in foodstuffs, supporting regulatory protocols while reducing operational interruptions in food production.

## Data Availability

The original contributions presented in the study are included in the article/supplementary material, further inquiries can be directed to the corresponding author.
